# The Theory of Value‐Based Payment Incentives and Their Application to Health Care

**DOI:** 10.1111/1475-6773.12408

**Published:** 2015-11-09

**Authors:** Douglas A. Conrad

**Affiliations:** ^1^ Magnuson Health Sciences Center University of Washington Seattle WA

**Keywords:** Incentives, agency theory, behavioral economics, value‐based payment

## Abstract

**Objectives:**

To present the implications of agency theory in microeconomics, augmented by behavioral economics, for different methods of value‐based payment in health care; and to derive a set of future research questions and policy recommendations based on that conceptual analysis.

**Data Sources:**

Original literature of agency theory, and secondarily behavioral economics, combined with applied research and empirical evidence on the application of those principles to value‐based payment.

**Study Design:**

Conceptual analysis and targeted review of theoretical research and empirical literature relevant to value‐based payment in health care.

**Principal Findings:**

Agency theory and secondarily behavioral economics have powerful implications for design of value‐based payment in health care. To achieve improved value—better patient experience, clinical quality, health outcomes, and lower costs of care—high‐powered incentives should directly target improved care processes, enhanced patient experience, and create achievable benchmarks for improved outcomes. Differing forms of value‐based payment (e.g., shared savings and risk, reference pricing, capitation, and bundled payment), coupled with adjunct incentives for quality and efficiency, can be tailored to different market conditions and organizational settings.

**Conclusions:**

Payment contracts that are “incentive compatible”—which directly encourage better care and reduced cost, mitigate gaming, and selectively induce clinically efficient providers to participate—will focus differentially on evidence‐based care processes, will right‐size and structure incentives to avoid crowd‐out of providers’ intrinsic motivation, and will align patient incentives with value. Future research should address the details of putting these and related principles into practice; further, by deploying these insights in payment design, policy makers will improve health care value for patients and purchasers.

This paper applies the economic theory of agency (and secondarily behavioral economics) to explicate the probable incentive effects of different models of “value‐based” payment in health care. Those forms of payment include recalibrated fee‐for‐service (FFS), bundled payment, capitation (global payment), and shared savings, coupled with adjunct performance incentives for quality and efficiency, including withhold and clawback provisions that shape ultimate incentive effects. “Value” is defined as maximum health benefit at minimum cost, and—operationally—better value translates into a combination of improved health outcomes and processes of care (clinical quality), better patient experience, and reduced costs of care.

This paper examines a number of market and nonmarket mechanisms for improving agency and, correspondingly, enhancing the power of value‐based payment. I describe the hierarchy of incentives, the differential power of group and individual incentives, and the importance of aligning external (payer‐to‐provider organization) incentives with intraorganizational provider compensation. The conceptual analysis is joined with targeted, directly relevant empirical evidence to derive a set of research questions and policy recommendations that follow from the theory and extant evidence.

## Purpose of the Paper

This paper is a conceptual exposition of the application of principal–agent theory to medical care, reinforced by evidence relevant to the application of agency theory in designing payment incentives for health care organizations and individual providers. It is not a systematic review, but instead integrates theory with empirical evidence most germane to value‐based payment. As the principal objective of the paper was conceptual, not empirical, my presentation of empirical evidence is necessarily selective, but intended to be a representative sampling of the existing literature.

Many of the concepts highlighted in this paper have been introduced previously in the theoretical and empirical literature (Dranove and White 1986; Ellis and McGuire 1993; Robinson 2001), so my purpose here was to offer a conceptual synthesis, illustrated by reference to available evidence, in one manuscript. That said, there are several novel ideas and applications in the paper. Examples include the following:

1. Conceptual argument for value‐based payment as a means of reducing the asymmetry of information between patients and providers;
2. Comprehensive reasoning in support of generally weighing evidence‐based process measures more highly than outcomes in provider incentive targets;
3. Theory‐based determinants of optimal incentive size, including considerations of potential crowd‐out of intrinsic motivation;
4. Implications from behavioral economics for (1) the role of loss aversion in reducing the optimal size of penalties and inducing greater participation of risk‐averse providers in two‐sided risk‐sharing contracts; (2) framing incentives as potential gains or losses to overcome status quo bias;
5. Logic for incentive targets primarily based on the individual provider's and the organization's own level of performance rather than peer rankings;
6. Implications of agency theory for design of incentive‐compatible contracts, and, in particular, the need to pay potentially more efficient providers and organizations an “information rent” to encourage them to participate and reveal their underlying efficiency;
7. Use of clawback provisions as an alternative to withholds as adjunct incentives for quality and efficiency;
8. Role of market mechanisms (e.g., competition, price, and quality transparency) in shaping the impact of value‐based payment on clinical quality, outcomes, and cost‐efficiency;
9. Effects of ownership and organization structure on value‐based incentive effects.

The section on future research questions identifies opportunities to advance knowledge regarding the impacts of value‐based payment incentives. Similarly, the policy recommendations reflect the implications of the author's analysis of theory and evidence relevant to the expected effects of value‐based payment.

## Motivation for the Choice of Agency Theory as a Guiding Conceptual Framework

Agency theory is well suited to contracting and other economic relationships between a principal and the agent(s). This theory can be applied to payment arrangements between the principal (e.g., the patient receiving care) and the agent (e.g., a provider organization). Optimal provider payment incentive design seeks to align the principal's and agent's interests.

**Figure 1:**
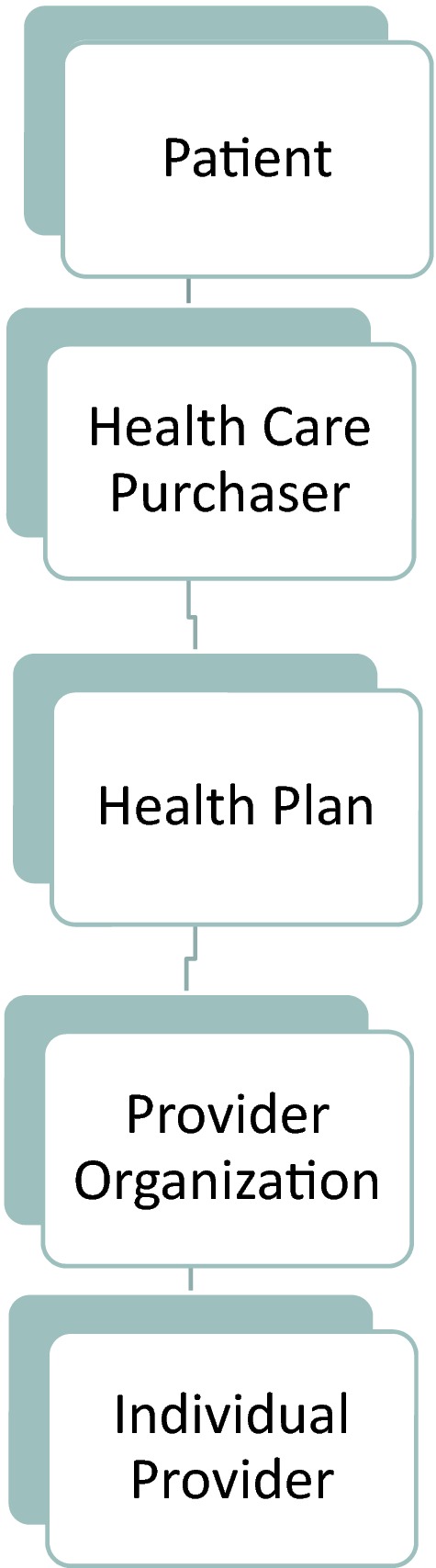
Principal–Agent Relationships in Health Care

One special feature of health care payment (see Figure 1) is the multiplicity and complexity of principal–agent relationships in health care (Casalino 2001).

The simplified description in Figure 1 illustrates that a provider organization or individual provider is an agent for the health insurer and for the patient. Similarly, the provider acts as agent for the provider organization contracting with or employing her. Payment incentive design ultimately must take into account the multiple agency connections inherent in payment for health services.

I assume that the desired provider behavior is “value”: maximum health benefit for the least total opportunity cost (including provider production costs, administrative costs, and patient opportunity costs). This term is equivalent to “economic efficiency” or net benefit. As dimensions of health benefit, quality of patient experience and ultimate health outcomes (evidence‐based care processes) are inherently valued, whereas clinical quality (following evidence‐based care processes) is an instrumental value—useful as a means to the end of improved health. A recent systematic review (Doyle, Lennox, and Bell 2013) concluded that patient experience was consistently positively related to outcomes across a broad array of health conditions, research designs, population groups, and settings. Minimizing the cost of producing health is the other aspect of “value,” and different incentive structures will appeal to different “principals,” depending on their particular weights on specific dimensions of value.

## Measurement of Value

Measurement is critical in designing provider payment based on value, rather than volume. Greater measurement error in assessing clinical quality, patient experience, and health outcomes suggests weakening the financial incentive applied to those dimensions of value—both size of incentive and weight of a given metric in the incentive structure. For example, if sample size of outcomes and particular behaviors for a particular provider or provider organization is small, precision of the estimate of health outcomes (e.g., blood pressure control for hypertensive patients) will be low, and the risk of false positives (rewarding good luck) or false negatives (penalizing bad luck) will be high. One way to mitigate this sample size problem is to lengthen the interval between observation and incentive, but that potentially weakens the timeliness and impact of measurement (i.e., the connection between the reward/penalty and the desired behavior or outcome). Valid, reliable, and timely measurement of each value dimension—costs, clinical quality (process of care), health outcomes, and patient experience—is necessary, but not sufficient, for success of value‐based payment.

Tradeoffs in designing value‐based health care provider compensation are more complex. The time lag between ultimate health outcomes and multiple influences (e.g., care from different providers, patient response, and comorbidities) beyond provider control implies that compensating providers on outcomes is potentially unreliable and unfair. This inherent measurement problem works against purely outcome‐based compensation. The asymmetry of quality, outcome, and price information between providers and health plan intermediaries and the patient or enrollee renders compensation based on clinical quality imperfect, but less so in terms of assessing the provider's actual contribution to patient health benefit. Finally, pure FFS payment stimulates quantity of service but fails to capture either quality of care or health outcomes.

As long as measurement of process quality and outcomes is unbiased in its design, and conducted with sufficiently large samples to produce relatively precise estimates of performance, prospects for value‐based payment seem positive. This suggests that a *mixed* payment model—based on measures of process quality, health outcomes, and transaction price measures—has the potential to improve the tradeoff between the higher relative measurement costs of paying on the basis of value and the potential patient health benefits of doing so. Optimal payment models will also be *context‐dependent*: baseline market conditions and structural relationships between purchasers, plans, provider organizations, and patients will vary geographically and over time, thus requiring a different mix of incentive size and structure.

In weighing use of process (clinical quality), patient experience, and health outcomes measures in incentive payment, the payer must balance the measurement costs of each performance attribute with relative benefits in terms of incentive effects on provider and patient behavior. I will argue that clinical quality and patient experience are more controllable by providers and provider organizations and measureable at relatively lower costs than outcomes (cf., Khullar et al. 2015). Therefore, the former ought to have higher weight in value‐based payments—provided that peer‐reviewed clinical evidence supports a strong link between the selected process indicators and health outcomes. To minimize the tendency toward “treating to the test,” process measures must be broad enough to capture clinical behaviors most strongly related to positive health outcomes (Houle et al. 2012).

Claims‐based measures are more readily available from transaction systems, but the measurement cost balance is shifting in favor of outcomes as meaningful use of electronic health records is expanding. In striking that balance, mixed payment systems achieve a better allocation of provider effort across different aspects of performance (Eggleston 2005). Chalkley and Khalil (2005) remark that if the payer can observe both outcomes and treatment quality (evidence‐based care processes), the optimal contract will be based on incentives for outcomes and clinical quality, subject to the costs of measuring both.

## Conceptual Framework

This paper's application of agency theory draws heavily on work of McGuire (2000, 2011) and several prior papers coauthored with other health economists (Conrad and Christianson 2004; Conrad and Perry 2009; Christianson and Conrad 2011; Conrad 2013). The basic idea is that the individual provider's objective is to maximize a combination of net income (minus the opportunity cost of physician effort) and patient health benefit, both of which are influenced by the quantity of service and quality of service. Quality is broadly defined to include patient experience, clinical quality, and health outcomes. Similarly, quantity is defined flexibly, and its operational specification will differ depending on payment method, for example, units of service under FFS, number of care episodes under bundled payment. Payers seek to design value‐based payment structures that induce providers to deliver the quantity and quality of service that will maximize the difference between patient health benefit and the total cost incurred for services received.

### Strong versus Weak Payment Incentives for Value

I distinguish between “strong” and “weak” payment incentives. Incentive strength refers to size of potential impact on provider net income and patient net health benefit and linkage between type of incentive (e.g., shared savings at the organization level, two‐sided risk organization payment models) and desired provider behavior and outcomes. Relatively stronger value‐based payment incentives will be larger *and* more directly tied to the payer's measure of expected value.

Financial incentive size must account both for the direct incentive effect, which elicits stronger response to those behaviors or outcomes that are relatively more rewarded or penalized relative to the alternative, and for diminishing marginal utility of net income, that is, the diminishing (but still positive) incremental value of net income to the provider. Diminishing marginal utility of net income implies that providers will be risk‐averse and will seek implicit or explicit “insurance” against the possibility of lower than expected net income.

Behavioral economics suggests that a penalty of given size for failing to advance health benefit will more strongly encourage providers to deliver improved health than an equally large reward for advancing health benefit. This economic reasoning is supported by experiments in psychology (Kahneman and Tversky 1979) and medicine (McNeil et al. 1982) that demonstrate greater incentive power of “loss aversion” compared to gain‐seeking.

Framing incentives as gains or losses relative to the status quo also increases the strength of response to a gain or loss of any given size. The provider's reference point matters. Recent work by Thaler and Sunstein (2009) highlights the power of “nudges” that frame decisions so that the default option (status quo) is inclined to be the one tending to increase value. An example would be reference pricing that favors the least costly treatment alternative of equivalent clinical effectiveness (Pearson and Bach 2010).

The size of value‐based incentive payments should cover prospective expected incremental costs of adjusting billing systems and internal clinical organization for the efficient practice, but not actual costs of every provider organization. This will require prospective estimation of efficient scale of practice and cost, and implies that a health plan or public program would pay for its share of the marginal transaction costs of revising practice organization to match the new structure of payment.

A third dimension of incentive strength is prospectivity and fixity of structure and size of payment. A prospectively fixed payment can be expected to elicit stronger behavioral response than a retrospective payment based on parameters not specified in advance. To illustrate this, it is sufficient to acknowledge that individual providers are predominantly risk‐averse. Hence, to induce them to participate in risk‐based contracts, the level of expected net income must exceed income under FFS payment, to compensate the individual provider for bearing actuarial risk (e.g., differences in population health beyond the provider's control during the performance period), as well as a certain degree of performance risk (e.g., adverse health outcomes that sometimes occur randomly even in situations for which the provider has exercised due care).

A corollary of this logic is that risk‐based payments (e.g., capitation and bundled payment) to larger provider organizations will be more sustainable, because those organizations will be more able to diversify individual provider‐level actuarial and performance risks. Given the incomplete evidence base for much of medical practice, providers and provider organizations face *diagnostic and treatment uncertainty*, which inherently cannot be diversified away by large numbers; this adds an additional layer of performance risk that suggests caution in implementing risk‐based payment. Provider organizations formed as partnerships, professional service corporations, and subchapter S corporations without outside, nonprovider owners will act *as if* they are risk‐averse to some extent, absent outside stockholders to diversify organizational risk.

A fourth factor driving incentive strength is duration and stability of payment arrangements. Microeconomics has established that long‐run price elasticities of supply and demand are both greater in absolute value than the respective short‐run elasticities. In the short run, certain resources are fixed (e.g., capital inputs) and this dampens supply response to demand price; similarly, one's knowledge of and the availability of substitutes are limited in the short run, which attenuates demand response to suppliers’ price signals (Silberberg 1990). This implies that both patient financial incentives and provider payment incentives will produce larger effects in the longer term.

Accordingly, greater stability of payment methods and certainty of payment levels over time will produce larger expected effect for a given level of payment. The Alternative Quality Contract in Massachusetts constitutes a successful example of this principle because of its 5‐year duration, the fixity of its global payment method, and the predictable evolution of level of PMPM payment over time (Chernew et al. 2001; Song et al. 2012, 2014). In contrast, payers unexpectedly lowering PMPM payments or unexpectedly raising performance thresholds in future periods, seeking to capture an increased share of savings, will find that they might have “shot themselves in the foot.” Short run surprises are likely to discourage future provider participation and to lower provider efficiency incentives. Similar consequences arise from Medicare accountable care organization (ACO) arrangements that inadvertently punish high‐performing providers by rewarding improvement in, but not the level of, performance.

One line of reasoning and two systematic evidence reviews (Conrad and Perry 2009; Van Herck et al. 2010) suggest that financial incentives based on individual provider or organizational performance relative to prospective, achievable benchmarks will induce stronger provider response to rewards and penalties than will relative performance incentives based on individual provider or provider organization rankings compared to a group of peers. Peers’ performance is not controllable, whereas the provider's own behavior is directly under his or her influence. This lack of control weakens incentive effects. Similarly, continuous provider progress toward improved value should be rewarded incrementally—rather than based an all‐or‐none threshold to qualify for any bonus payments for quality or outcomes. Selecting clear, achievable performance targets strengthens incentives for quality improvement (Kiefe et al. 2001; Kirschner et al. 2012).

However, relative performance incentives still might belong within a mixed payment system (Prendergast 1999). Incentives based on performance relative to peers partially filter out common environmental and market factors that otherwise confound providers’ attainment of performance targets. Moreover, relative incentives can be implemented predictably within a fixed payer budget, whereas, if a larger than expected number of providers achieve targets for incentive bonuses under an absolute incentive regime, the payer would suffer losses to the incentive pool. The single peer‐reviewed study of relative performance incentives (Young et al. 2007) did not find significant effects on physician performance.

On balance, payment subject to individual provider and organization‐based, achievable performance standards offers stronger incentives (cf., Mullen, Frank, and Rosenthal 2010). Budgetary uncertainty could be mitigated by announcing a schedule of reduced payments over time for providers in proportion to any shortfall relative to achievable performance targets. In addition to tapping providers’ loss aversion, this approach fits findings from the literature (Rosenthal and Dudley 2007; Conrad and Perry 2009; Van Herck et al. 2010) regarding dominance of absolute performance standards in P4P programs. In theory, the incentive power of loss aversion is greater than that of gain‐seeking; in practice, penalties should be introduced carefully as extant evidence implies greater positive impacts of rewards than penalties on performance (Conrad and Perry 2009; Van Herck et al. 2010). A combination of incentives for improvement and for level of achievement likely offers the greatest provider motivation within an absolute incentive regime (Petersen et al. 2006; Van Herck et al. 2010).

The strength of financial incentives also has the potential to substitute external motivation for the internal, professionally driven motivation of health professionals and the provider organizations in which they work. The next section deals with this topic, which sets the stage for discussing the design of a menu of value‐based incentive contracts.

### Nonfinancial Incentives and Potential Crowd‐Out

The main emphasis in this paper is on extrinsic financial incentives, but nonfinancial incentives must be considered in parallel, for example, reputation or brand, intrinsic motivation, and altruism. Will the use of financial incentives as extrinsic motivators tend to “crowd‐out” intrinsic motivation? Put another way, will use of strong financial incentives tend to reduce providers’ commitment to professional norms and their inherent motivation to act first and foremost in patients’ interest (Benzer et al. 2013; Congleton 1991; Deci, Koestner, and Ryan 1999)?

The answer to the “crowd‐out” question depends on whether patient health benefit and provider net income substitute for or complement one another in the provider's objective function. Payment level and method are major determinants of net income, so payment according to value would tend to drive patient health benefit and provider net income in the same direction. Thus, rather than inducing crowd‐out, value‐based incentives would tend to make patient health benefit and provider net income complementary objectives. A systematic literature review of pay for performance (P4P) (Van Herck et al. 2010) indicates that involving providers in program development, implementation, and evaluation is associated with positive effects of P4P, and effect sizes are greater than in programs not engaging providers. This form of “soft autonomy” tends to encourage provider buy‐in to financial incentives.

There is some evidence from the experimental economics literature that any crowd‐out of intrinsic motivation is more likely in retrospective FFS or FFS with P4P incentive arrangements than in salary systems, capitation with report card, or capitation with P4P (Green 2013). Provider loss aversion also implies that penalties for underperformance should not be so large as to trigger a counterreaction among providers seeking to replace lost income by inducing additional demand for fee‐based services or skimping on necessary, but difficult‐to‐measure services.

### Developing a Menu of Value‐Based Payment Contracts

Having addressed the applicability of strong versus weak incentives, I now address the role of a menu of contracts in improving value in this section. Incentives must be designed to address two inherent challenges due to conflict between interests of principal and agent: the “hidden information problem,” which gives rise to adverse selection; and the “hidden action problem,” which is the root cause of moral hazard in incentive contracting (Laffont and Martimort 2002). I have addressed the problem of hidden actions of providers and consumers, that is, supply‐side and demand‐side moral hazard. I next briefly discuss design of payment incentives that address payers’ hidden information (adverse provider selection) problem.

The payer's response to these challenges is to fashion a *menu* of “incentive‐compatible” contracts, which induce agents to reveal their true types (i.e., the provider's actual efficiency and skill in performing any given set of tasks), thereby reducing asymmetry of information between payer and individual provider or provider organization. Incentive‐compatible contracts between principal and agent must also satisfy the “participation constraint”—that is, to accept the contract, the agent must be at least as well off as under the next best available alternative contract. Thus, the size of payment incentives should be sufficient to yield an expected (but not guaranteed) competitive return on equity to the individual provider or provider organization.

Given incomplete and asymmetric information, the principal's contract with the agent must generally pay an “information rent” to the more efficient agent: the provider with greater skill who can, by definition, produce more of the principal's desired output with less effort. This information rent can be thought of as the excess payment above the efficient provider's marginal cost—to induce that clinician not to pretend to be a less efficient one and to hold out for a higher level of payment. For example, the physician with greater clinical skill will produce improved health outcomes and care processes with less time, effort, and practice inputs than will a less skillful clinician. Thus, higher quality providers will be differentially attracted to value‐based contracts.

Payers face a tradeoff between productive efficiency of, and excess payment to, providers. Optimal contracts under conditions of asymmetric information can be characterized as follows:

- More efficient providers supply the optimal output desired by the payer; the payer accepts a below‐optimum level of output from less efficient providers.
- Only the efficient providers capture an excess payment, which equals the difference between the marginal productivity of the efficient and inefficient providers, multiplied by the below‐optimal level of output induced from less efficient providers.

Another situation emerges wherein the payer decides to “shut down” any contracting with the less efficient providers. In that case, no excess payment is offered to more efficient (higher quality) providers, but a below‐optimal level of output is supplied, given absence of production by less efficient providers. The payer has traded off some value to reduce the size of payment to higher quality providers. The inefficiency in this case equals the forgone health value and is solely attributable to asymmetry of information between the principal and agents (Laffont and Martimort 2002). Exclusion of, or reduced plan payment for, some providers, is an example of health plans’ use of “narrow networks” of participating providers (Corlette et al. 2014). Selected payers are steering patients toward centers of excellence (another term for narrow networks) of low‐price and high‐quality providers by the use of reference pricing, which requires the patient to pay the marginal cost for providers whose fees exceed the plan's allowed amount (Robinson and MacPherson 2012).

### Effects of Ownership and Organizational Form on Payment Design

Optimal provider payment incentives are shaped by nuances of ownership (i.e., for‐profit vs. not‐for‐profit for hospitals) and provider organizational form (e.g., integrated medical group vs. independent practice association [IPA]). Ownership is an important financial incentive, in that owners capture their share of organization net income and are motivated to pursue maximum profit through cost minimization and strategic pricing. The implication for payers is that not‐for‐profit providers will have weaker incentives than for‐profits to hold out for above‐competitive prices, which will affect the *level* of contracted prices, but not necessarily the method of provider payment. A study by Lynk (1995) found that while mergers between private for‐profit hospitals led to increased prices, those between not‐for‐profits were followed by reduced prices. Dranove (1988) outlines the theory and certain empirical support behind the argument that, other that, other things equal, not‐for‐profits will price at lower levels than for‐profits.

Organizational form influences incentive design. Guterman et al. (2009) present the case for aligning payment form with organizational form: taking into account the capacity of the organization to accept *actuarial risk* (variation in health beyond the control of the provider organization and individual provider) and inducing the individual provider and organization to minimize *performance risk* (variation in health due to provider errors: undertreatment, overtreatment, or mistreatment). Table 1 illustrates my extension of this reasoning.

**Table 1:** Aligning Incentives with Organization Form

Organization Form	Aligned Value‐Based Incentive Design
Small, independent practices	FFS with risk‐adjusted value incentives
Primary care groups	Risk‐adjusted primary care capitation with adjunct incentives
Single‐specialty groups	Risk‐adjusted “contact capitation” with adjunct incentives
Hospitals	Case rates extending DRGs to episode of care and variants of bundled payment
Multispecialty groups, IPAs, and integrated delivery systems	Risk‐adjusted global cap with adjunct incentives
ACOs	Risk‐adjusted global cap with adjunct incentives or “equivalent” two‐sided shared savings

The basic idea is that small independent medical practices are poorly equipped to bear significant actuarial risk for random variation in health status. Consequently, FFS arrangements will be most feasible for them, potentially tied to adjunct pay‐for‐performance (P4P) incentives based on measures of clinical quality and patient experience, but not to health outcomes or total cost per patient over time—reasoning that the smaller sample size would imply greater random variation in average cost and health outcomes.

Put another way, small practices do not benefit from the “law of large numbers.” By the nature of their practice structure, small independent practices face greater costs of coordinating care for patients across different clinical conditions, which militates against placing them at risk for total care costs per person. Thus, risk‐adjusted primary care capitation, including withholds structured to guard against excess specialty referrals plus value‐based adjunct incentives, aligns well for these organizations. Paying single‐specialty groups per episode per referred patient (“contact capitation”) plus adjunct value incentives also discourages excess servicing and encourages value.

Building on the base of diagnosis‐related group (DRG) payments, hospitals are poised to move to case rates for episodes of care, joined with adjunct value‐based incentives. Because of scale and internal capacity for cross‐specialty care coordination, larger multispecialty groups, integrated delivery systems, and ACOs are better positioned to accept risk under bundled payment and risk‐adjusted capitation, respectively. Depending on the extent of interpractice coordination of patient care protocols, information systems and contracting (IPAs) also have these capabilities. Ownership and organizational form thus significantly influence payment incentive design and the impact of financial incentives. In addition, market mechanisms also have the potential to significantly improve the agency between payers and their enrollees.

### Market Mechanisms for Improving Agency between Payers and Enrollees

Two mechanisms play a particularly important role in more closely aligning interests of payers and health plan enrollees. One is improved, more symmetric information on quality, outcomes, and prices relevant to health plans, their enrollees, and providers. As individual enrollees tend to be at an informational disadvantage relative to both health plans and providers, a health plan that offers publicly available, population‐based information on actual transaction prices (more valuable than “billed charges”), quality, and outcomes effectively narrows the informational gap between enrollees and providers.

A second market mechanism—more effective competition within the markets for health care delivery (providers), health insurance (the plans), and integrated health plan‐provider organizations—also would improve agency of those intermediaries on behalf of their patients and enrollees. To the extent individual consumers are less attentive to such information—either because of first‐dollar health insurance benefit designs, loyalty to their current providers, or poor health care literacy—organized purchasers would have an interest in doing this on behalf of their workers, enrollees, and beneficiaries. However, given enrollee turnover and failure to assure uniform and unbiased information disclosure among competing insurers, there is a “public goods” (externality) problem with voluntary disclosure. The insurer disclosing such information may not capture the benefits in cost, quality, or outcomes for the enrollees on whose behalf it originally invested. Similarly, asymmetric or biased information between the plan and its enrollees—by raising consumer search costs among alternative plans—can enhance a given plan's market power.

Either government or an otherwise neutral organization is required to design and enforce these price and quality transparency mechanisms. Only government or a quasi‐governmental public–private partnership underpinned by organizational public commitments can monitor and regulate provision of “public goods,” such as common investments in public health information infrastructure; agreements on quality, outcomes, and patient experience measurement; and value‐based payment methods (not levels of payment).

State action also has the capacity to create safe harbors (antitrust immunity) for collective agreement on payment method. As Enthoven (1993) has argued, effective competition on value requires managing rules of fairness, health plan participation, price and quality disclosure, and enrollment, as well as guidance toward unbiased risk selection and plan design that will encourage patient demand sensitive to price and quality. Health insurance exchanges enabled by the Affordable Care Act are a potentially strong step toward managed competition (Aaron and Lucia 2013; Barnes, Hanoch, and Rice 2014). Reduced barriers to entry for health plans and effective antitrust policy and implementation are key ingredients in perfecting the agency role of health plans.

A third market mechanism shaping agency is the intermediary role of public purchasers (e.g., Centers for Medicare and Medicaid Services [CMS]), private insurers, and employer–purchasers relative to their beneficiaries and enrollees. Public purchasers and private insurers will tend implicitly to weigh clinical quality, patient experience, and health outcomes less than direct patient beneficiaries of those components of value, whereas claims costs are a direct hit to public budgets. Moreover, because patients bear directly the opportunity costs of travel time and delays of treatment and enjoy the benefits of positive health outcomes and supportive caring patients, they will place greater weight on quality of experience and health outcomes than their insurer or public purchaser.

Employer–purchasers have particularly well‐aligned incentives on behalf of *current* employees’ health and health care costs because, in a competitive labor market, improvements in value of employees’ health benefit package will be reflected in lower wage levels than would otherwise be demanded by employees: the employer fixes total compensation level, comprised of wages and employee benefits, so a rise in value of one component leads to a decrease in the other. This “compensating differential” has been demonstrated convincingly in health care (Gruber and Krueger 1991; Gruber 1994; Madrian 2006; Emanuel and Fuchs 2008).

## Implications for Choice of Value‐Based Payment Methods

*FFS payment* creates strong provider incentives for higher volume, particularly for those services and procedures with higher net income margins per unit of service. Under FFS, the provider is at risk only for unit costs of services, not for patient health or total treatment costs. Therefore, optimal value‐based provider payment design would assign higher fees to those services that produce greatest patient benefit in health outcome, clinical quality, and patient experience. Size of FFS payment (which influences net income margin per unit of service) can be altered to influence volume incentives; specifically, if the margin on each service is equal and positive, the potential incentive for providers to “induce” demand for more profitable services is eliminated (McGuire 2011). This form of recalibrated FFS could fit into a value‐based payment portfolio.

In setting those fees, the FFS payer acting as the perfect agent for the patient should strive to achieve provider *financial neutrality* across services—setting net income margins equal across services, to the extent possible. This means setting prospective payment per unit of service equal to the best estimate of the efficient practice's marginal cost of production and to marginal health benefit for the patient. Over time, less efficient providers will cease production of services paid below their marginal costs. The administrative and informational costs of designing FFS payments with this efficiency property are one practical argument against basing a value‐based system on FFS.

Patient copayments through value‐based insurance design (VBID) are unlikely to solve incentive problems with FFS because of the considerable asymmetry of information between providers and patients. Three corollaries emerge from this logic:

- Public policy should invest in serious cost accounting for both hospitals and medical groups to better measure the production costs of different services. Such activity‐based, fully allocated cost systems exist in the private sector for hospitals, and a revised, updated resource‐based relative value (RBRVS) schedule could be applied as a baseline for costing units of professional services, for example, for physicians and medical groups.
- Policy makers should deploy comparative effectiveness research to determine the marginal health benefits of different services. That comparative effectiveness research is directly applicable to VBID and should be deployed beyond the current emphasis on pharmaceuticals. Setting unit prices close to the unit cost of services solves the problem of “demand inducement.”
- If FFS payments were based on equating marginal expected health benefit with marginal cost per service, the provider would be induced to provide optimal value per service, but not to choose the optimal service mix per episode of illness or over calendar time (e.g., the year). In that sense, FFS is best suited to acute, one‐time health problems.

*Bundled, or episode‐based, payment* implicitly assumes that the objective of the principal (patient or the health plan/purchaser acting on his or her behalf) is to maximize value from health care provision per episode of illness. The payer's definition of episode of care attempts to match the period of care with a clinically accurate period of the actual illness. Bundled payment places providers at risk for efficient care (often termed “performance risk”) of the patient during an episode of care. If the bundle includes multiple providers involved in provision of care during the episode, an added complication is articulating a payment sharing arrangement among the providers. Bundled payment incents cost reduction per episode, but one payer concern would be “unbundling” of care or repeated care episodes (analogous to hospital readmissions), particularly for procedure‐based care, for example, care episodes triggered by hospitalization for surgery. Unbundling might be addressed best by broad definition of an episode that includes preintervention (work‐up), intervention, and postintervention (follow‐up) periods, subject to adjunct incentives for total cost of care (TCOC) savings.

To some extent, clinically appropriate classification of the episode adjusts for case mix variation and thus eliminates random, uncompensated variation in actuarial risk (health differences) across patients in the provider's panel. Another decision in bundled payment design is the extent to which the expected cost of potentially avoidable complications (PACs) is built into the prospective payment per episode. For example, the PROMETHEUS episode‐based system includes 50 percent of the expected cost of PACs in its prospective rates (DeBrantes, Rastogi, and Painter 2010); this partially weakens the incentive for “value,” both by covering some potentially unnecessary costs and by not holding providers fully financially accountable for providing optimal health benefit to the patient.

Implementing bundled payment has proven quite difficult, and many of those practical challenges reflect its conceptual limitations. Hussey, Ridgely, and Rosenthal (2011) observed a host of problems in the PROMETHEUS pilots: difficulties in defining the bundle (e.g., tying episodes of care to underlying episodes of illness, as opposed to an arbitrary time period); dealing with comorbid conditions; allocating accountability among distinct providers; and others. The Integrated Healthcare Association bundled payment demonstration encountered similar challenges (Ridgely et al. 2014). It is likely that bundled payment will be implemented primarily for acute care episodes and procedure‐based care, as chronic conditions are more naturally addressed by a person‐centered global payment.

*Capitation, or global payment* (per person), places individual providers and provider organizations at greatest risk for cost variation. Providers bear both unit cost and volume risk for number and type of services per episode of care and for number of episodes over time. If better clinical (process) quality and patient experience reduce the number of care episodes and of additional services per episode, capitation offers the strongest positive incentive for health benefit if payments are “risk‐adjusted,” that is, pegged at higher levels for inherently more complex patients. Also, as payment is per person, provider organizations have the strongest incentive to accept more patients under this regime. Capitation is not a strong direct incentive for value unless capitation size is explicitly tied to expected patient health benefit (through risk adjustment and incentives for evidence‐based practice) and offers a large enough margin over cost to induce providers to attract new patients based on value. Use of a P4P quality incentive as an adjunct to the base, risk‐adjusted prospective capitation payment would also mitigate potential stinting in quality of care.

*Shared savings* incentive models typically blend prospective FFS with sharing of retrospective, TCOC savings between payer and provider organization. By sharing in any total cost savings, the payer's intent is to attenuate providers’ potential tendency under FFS to overtreat and overprescribe. The strength of the incentive increases with the provider's share of any retrospective TCOC savings and with the level of TCOC savings expected by the provider. These shared savings models could be one‐sided (upside potential, savings only, no downside risk for provider deficits relative to the predetermined TCOC budget), or two‐sided (both shared savings and shared downside risk). Schmidt and Emanuel (2014) delineate an innovative notion: how “inclusive shared savings” (for *patients*, as well as providers and payers) might be deployed to strengthen incentives for cost‐efficiency, and therefore improved value.

Given the greater power of a loss compared to a gain of equal magnitude (Kahneman and Tversky 1979), payers could achieve the greatest incentive effect by setting provider share of losses (deficits against the TCOC target) somewhat lower than the provider share of gains (TCOC savings). That incentive design would have the double benefit for payers of increasing participation of risk‐averse and loss‐averse providers and minimizing size of the required incentive. Two‐sided models would have stronger incentive effects than shared savings alone. FFS payment for evaluation and management services and evidence‐based preventive screening would strongly support value, while maintaining TCOC savings incentives. By adding outcome‐ and quality‐based P4P incentives to shared savings, payers could achieve a stronger effect on value.

The greatest potential challenges to payers in shared savings models are the twin requirements to choose a level of TCOC budget per insured member per month (PMPM) that offers strong incentives for total savings, and to craft a valid and reliable method of attributing patients to the providers accepting payment based on shared savings. Such models assume that shared savings will derive mainly from reduced hospitalization and specialty costs, which are only partially under the control of the referring primary care provider (PCP). Often shared savings targets are tied either to overall PMPM costs in the local market or to those of another comparison group of provider organizations. Accordingly, the power of the shared savings incentive will depend on providers’ perceptions of validity and reliability of patient attribution and of TCOC performance targets.

The other major challenge of shared savings models is that the predominant “frame” for payment is FFS, with retrospective “settling up” to determine whether savings have been achieved. Behavioral economics have shown that status quo bias and framing context are crucial determinants of decision making (Kahneman, Knetsch, and Thaler 1991). Shared savings incentives also are weakened by the delay in retrospective determination of cost savings.

### Adjunct Financial Incentives to Support Value‐Based Payment

P4P incentives and shared savings (based on TCOC) can be deployed to offset some limitations of FFS as a general method for inducing value improvement. Withhold and “clawback” provisions also can be deployed to encourage value over volume. Withholds can be deployed to incent TCOC savings over the defined period or to return withheld payments in proportion to attainment of prospectively established quality or outcome targets. Clawback provisions retract a portion of previous payments over the performance period, based on retrospective determination of the provider's failure to achieve prospective cost, quality, or outcome objectives.

Both provisions shift performance risk to providers; while the withhold's impact is spread over time, clawback provisions are executed only at the end of the performance period. Application of such a provision represents a loss of provider dollars. For payers imposing a clawback to achieve an incentive effect equal to that of a withhold, expected clawback size must balance the present value loss to the payer of deferring the clawback with the greater incentive power of the clawback due to provider loss aversion. While withhold and clawback provisions are generally tied to cost savings, they could be applied to stimulate improvements in care process and patient health outcomes by linkage to clinical performance. In that sense, quality‐ or outcome‐based withholds would be particularly useful adjuncts to episode‐based bundled payment or capitation incentives, for which concerns about stinting of care are more prominent than FFS.

## The Hierarchy of Incentives

Provision of health care is a “team sport” requiring coordination between incentives and behavior of the group (or team) and the individual. Achieving the optimal balance between these incentives requires balancing cooperation and coordination among group members and strong individual‐level incentives. Group incentives are subject to a “public goods” problem: individual members may free‐ride on performance of other group members unless selective, individual‐level performance measures are applied to distribute a share of group incentive payment in proportion to individual provider contribution to the team's production (Olson 1965). Extant systematic reviews (Conrad and Perry 2009; Van Herck et al. 2010), as well as a recent randomized trial of group‐ versus individual‐level physician incentives (Petersen et al. 2013), support the superior strength of individual‐level bonuses.

The weight of evidence and theory supports use of group‐level incentives to build improved information and care management infrastructure, rather than as a motivator for individual clinicians. One upside of group‐level incentives is that the actuarial and performance risk of unmeasured variation across team members in patient case mix and random variation in cost, quality, and outcomes is diversified. This allows the payer to apply stronger incentives dollar‐for‐dollar to risk‐averse providers acting as a group.

Evidence from the contracts and labor economics literature strongly suggests that team‐based pay (such as fixed salary) is associated with lower average productivity and wider variance in output (Lazear 2000). While team‐based pay also attracts employees of medium ability, its constraints and peer pressure discourage employees of above‐average and below‐average ability (Weiss 1987). The challenge for health care organizations is to craft team‐based pay that rewards care coordination and cooperation, while encouraging individual effort to produce maximal value for the team's panel of patients.

Group incentives will be more appropriate and sustainable for members of physician partnerships and integrated medical groups that share revenue, expense, and financial risk and are capable of coordinating patient care and clinical and economic information. IPAs and provider networks might be equally capable of sharing financial risk through value‐based capitation, bundled payment, mixed models, and shared savings. However, potentially higher costs of coordinating care and information among independent practices imply that individual provider‐level incentives will be necessary to augment group incentives. For similar reasons, individual‐level payment incentives should receive greater weight as group size increases (Gaynor and Gertler 1995). High‐powered individual incentives for physician productivity (e.g., relative value unit‐based compensation) are best financially aligned for medical groups under predominantly FFS contracts with health plans. This alignment between incentives of external contracts between the group and payers and individual compensation incentives becomes increasingly important as group size increases.

### Aligning Incentives between External Payment and Internal Compensation

Continued reliance on FFS would defeat movement toward value‐based payment. Consequently, more relevant examples of aligning external plan‐to‐organization payment with internal compensation incentives are those in which health plans are paying the organization on shared savings, bundled payment, or global payment (capitation). The core concept is to match internal compensation structure with external payment methods. Consider the following examples, in ascending order of strength of value‐based, person‐centered internal incentives (illustrated in Table 2):

- For medical groups predominantly paid on FFS, with adjunct value‐based performance incentives, the preponderance of provider compensation would be production‐based, with adjunct incentives tied to value‐based parameters and in proportion to total group payment based on value.
- With bundled payment per episode, well‐aligned internal compensation arrangements for participating providers (e.g., hospital, specialists, and PCPs) would look like this: severity‐adjusted DRG rates for hospitals and disincentives for readmissions, risk‐adjusted per episode payment to specialty providers (sometimes referred to as “contact capitation”), and salary compensation for PCPs—with all providers sharing in value‐based (TCOC, quality, and outcome) incentives.
- Under health plan, risk‐adjusted capitation payments to an ACO, internal compensation structure for participating specialists, and PCPs, respectively, would be very similar to that for episode of care, bundled payment. The main differences would be that salary compensation for PCPs would include an adjunct incentive for risk‐adjusted panel size.

**Table 2:** Matching External Incentives with Internal Compensation Arrangements

External Incentives to Organization	Internal Compensation
FFS with adjunct value incentives, including care coordination PMPM	Production‐based with proportionate adjunct incentives for value (“Triple Aim”)
Risk‐adjusted, bundled, episode of care payment	Severity‐adjusted hospital case rates; contact capitation for specialists; PCPs on fixed salary with adjunct value incentives
Risk‐adjusted global payment (capitation)	Fixed salary with adjunct value incentives

Existing empirical evidence is directionally consistent with these theoretical implications. Robinson et al. (2009) found that large medical groups subject to health plan P4P incentives based on quality and satisfaction were more likely to compensate their PCPs similarly. Groups predominantly paid on capitation were more likely to compensate their physicians on salary, rather than production. An earlier study concluded that medical groups and IPAs in markets with more managed care were less likely to base physician compensation on production (Robinson et al. 2004).

## Research Questions Raised by the Framework

*Which areas and levels of medical care are best suited to strong incentives?* One might plausibly argue that—because of difficulty of accurately measuring outcomes and physician quality, given small sample sizes—high‐powered incentives might be less effective for individual providers. Defining effective measures of health risk and case mix can ameliorate this problem, but it has practical limitations. Larger provider organizations (hospitals and multispecialty groups) are more capable of diversifying population health risk and managing and coordinating the care of populations, which should position them to bear health risk and manage care under high‐powered incentive contracts (value‐based and global capitation payments, for example).

As individual providers, whether primary care or nonprimary care specialists, are particularly well‐positioned to influence patient experience, value‐based adjunct compensation incentives should place strong emphasis on patient experience. The experience dimensions of continuity of care, coordination of care, communication with the patient, shared decision making, communication among providers, access, and timeliness merit particular attention.

*What kind of incentives will work best for ACOs?* ACOs are new, “virtual” forms of clinically and economically accountable entities attempting to replicate the cost‐efficiency, care coordination, and clinical quality advantages of vertically integrated, prepaid group practice. Their emergence raises several questions for research:

- What contract types are best for the ACO as umbrella entity, and which incentives will encourage the most clinically effective and economically efficient performance?
- Can ACOs borrow from successful practices in group model HMOs that unify the care continuum under one organizational umbrella?

*What practical incentive strategies will work best to mitigate gaming by different types of organizations? To what extent are regulatory, informational, and organizational designs likely to perform better than incentives in preventing gaming, risk selection, and “teaching to the test”?* Regarding the latter, to what extent are high‐powered financial incentives inherently incompatible with building an organizational culture that supports altruism and intrinsic motivation among clinical professionals and other members of the health care team?

*What payment incentives best promote value and provider participation?* Agency theory stresses appropriate design of incentives to solve adverse selection problems due to asymmetric information between patients and providers (e.g., small practices self‐selecting into FFS payment regimes that offer weak incentives for value, but which are more feasible for independent practices). Scott et al. (2011) observed that none of their reviewed studies addresses physician selection into or out of incentive arrangements. DeBrantes and D'Andrea (2009) found that amount of potential bonus was a strong predictor of physician participation in the Bridges to Excellence incentive program. Additional research regarding determinants, and mix, of provider participation in incentive programs is a high priority.

- Are there feasible value‐based payment contracts that would offer a sufficient financial return to risk‐averse providers in small practices to induce them to participate, yet would offer stronger incentives for producing value? For example, could recalibrated FFS (with fees set to approximate efficient marginal cost), joined with P4P quality and efficiency incentives, replicate the cost‐efficiency of risk‐adjusted capitation, without potential stinting on care?
- What forms of value‐based contracts might replace the current encounter‐based and cost‐based payment arrangements of rural health clinics and critical access hospitals? Are regionally based ACOs a feasible structure for smaller rural providers to maintain population health collectively with virtual care coordination and health information exchanges? What type of entity is best structured to serve as the ACO's focal organization?
- What specific variables (e.g., market conditions, provider specialty, other provider characteristics, patient mix) explain why certain providers and provider organizations are willing to accept risk‐bearing, value‐based payment, while others prefer FFS? Prendergast (1999) highlights the need to identify “instruments” (exogenous variables beyond the provider's immediate control that are correlated with the choice of payment incentive, but not with the results of interest). To estimate the effect of different payment incentives, researchers must disentangle the impact of self‐selection versus payment incentives.

*What new forms of shared savings arrangements are likely to reduce total costs of care over time?*

- How does “sharing ratio” (health plan vs. provider share) affect success of these arrangements in reducing cost without compromising quality? More fundamentally, do shared savings arrangements actually achieve cost reductions, or is the shared saving incentive simply not visible enough at the point of care to influence clinical behavior?

*Is there a “sweet spot” along the spectrum of risk‐bearing at which the comparative advantage of payers in bearing actuarial risk is balanced with the ability of individual providers and their provider organizations to manage performance risk?* What mixed payment schemes would be optimal (Frakt and Mayes 2012)?

*What payment design and information support mechanisms are most likely to produce favorable results for CMS?* Of the four Medicare demonstrations of value‐based payment, only one (the Participating Heart Center Bypass Center Demonstration of bundled payment) achieved significant savings, with little on effect of patient outcomes. Among the others (all based on shared savings models), the Physician Group Practice Demonstration showed little or no effect on Medicare expenditures and small improvement in care processes. Similar results were obtained for the Premier Hospital Quality Incentive Demonstration and the Medicare Home Health Pay for Performance Demonstration (Nelson 2012). These studies would require a mix of qualitative and quantitative analyses to discern underlying implementation challenges for value‐based payment reform (Conrad et al. 2014).

*To what extent are extrinsic financial incentives likely to crowd‐out intrinsic motivation?*

Once a financial incentive for performance has been implemented and running for some time, to what extent might provider performance revert to preincentive levels or even below if the incentive is withdrawn? Petersen et al. (2013) noted that the positive effect of an individual physician incentive did not continue in the postincentive period. However, as postincentive performance did not drop below baseline, these findings are not evidence for crowd‐out *per se*.

## Policy Recommendations

Agency theory implies that strong incentives for value (i.e., health benefit relative to cost) should be fashioned within provider payment arrangements. Well‐designed practical trials and controlled before‐and‐after observational studies of different payment regimes in different settings should be conducted. These studies should evaluate such models as recalibrated FFS, bundled episode‐based payment, risk‐adjusted capitation, and shared savings arrangements in different organizational settings. In spite of the significant resources required to obtain efficient (precise) and unbiased estimates of practice costs, outcomes, and clinical quality, future studies of value‐based payment should incorporate all those metrics to paint a complete picture.

Fee‐for‐service payment is likely to continue in the ongoing transition from “volume to value.” In that interim period, I recommend that private and public payers set FFS prices equal to their best estimate of efficient average production costs (beginning with DRG‐based prices per discharge for hospitals and RBRVS‐determined prices for physician services), including an estimate of the average risk‐adjusted return on equity for the provider.

Allowing providers to vary their prices patient‐by‐patient (based on perceived marginal benefit to the individual patient) would be tantamount to price discrimination. Given imperfect competition among providers and asymmetric information between providers and patients regarding price and quality, individualized pricing patient‐by‐patient is inadvisable. In the limit, such “perfect” price discrimination by providers, while theoretically achieving an optimal level of output, would extract all consumer surplus from payers and patients and transfer those dollars to providers. Such a regime would approximate the usual, customary, and reasonable regime of physician pricing that predated the adoption of RBRVS.

Instead, I recommend a form of *reference pricing*, in which the external payer sets an estimated cost‐based price for the least costly, clinically equivalent service among different treatment options. This approach would require data from comparative effectiveness studies, which Pearson and Bach refer to in their 2010 article as “paying equally for services that provide equivalent results” (p. 1798). They discuss the case of different treatment options for low‐risk prostate cancer. For example, intensity‐modulated radiation therapy was reimbursed by Medicare at $42,000, compared to approximately $10,000 for traditional, three‐dimensional radiation therapy—without evidence regarding the comparative clinical effectiveness or potential toxicities of the alternate treatments (pp. 1799–1800).

There is no clear‐cut line of demarcation between areas appropriate versus inappropriate for applying strong incentives. Nevertheless, theory and evidence both suggest that small provider organizations are not equipped to assume actuarial risk. Random variation in population health is best borne by insurers. Well‐validated, person‐level health risk adjustment measures are a critical component of value‐based payment, so that all provider organizations are only bearing performance risk. Smaller provider organizations can band together in IPAs for risk contracting and economies of scale in management, information systems, and quality improvement initiatives, but whether such virtual integration can replicate the benefits of vertical integration and unified ownership remains an open question (Mehrotra, Epstein, and Rosenthal 2006; Rodriguez et al. 2009).

Agency theory offers strong guidance to policy makers on the targeting of strong (high‐powered) incentives. The richest source of its explanatory power is focusing attention on the size and form of financial incentives required to induce participation by providers, as well as the elements of incentive contract design that will mitigate supply‐side moral hazard and adverse selection by providers. Future theoretical research and empirical testing should address the forms of per person, per episode, and per service payment and adjunct incentives (e.g., P4P, withholds, clawbacks, shared savings) that result in superior outcomes and costs of care. Because incentives are not the whole game, future research should also explore the types of performance reporting, incentive measures, and regulatory oversight of market performance generate the best results—and under what circumstances.

Mullen and colleagues’ study (2010) of the PacifiCare and IHA P4P programs in California did not find significant evidence of positive spillover effects on quality from rewarded to unrewarded measures. While raising concerns about “treating to the test” through a possible shift in provider efforts to lower time‐ and effort‐intensive activities, their findings did not offer definitive evidence of this phenomenon. This study should be replicated in other markets and with multipayer participation.

Strong pressure should be exerted by purchasers to encourage price and quality transparency and value‐based benefit design, and to eradicate barriers such as outdated, FFS‐based transactional systems and lack of transparency of total expected premium plus out‐of‐pocket costs under different health plans. The reasoning behind this policy recommendation is that strong provider payment incentives—absent supporting moves to reduce opacity of price, quality, and outcomes data and to align benefit design with value—are unlikely to achieve their full potential in improving value for patients (and their intermediaries: public and private purchasers and health plans).

Deriving valid and reliable measures of value—a necessary antecedent to implementing value‐based payment—requires continued public investment of resources, especially given the public goods nature of information. Metrics developed by HEDIS, ongoing efforts at development of consensus quality and outcome measures by the National Quality Forum, patient experience survey items imbedded in CG‐CAHPS and other data collection metrics should be integrated within a broad national strategy for value‐based payment, underpinned by a scientific and professional consensus. Coordinated and sustained value‐based measurement and payment by CMS, the Veterans Health Administration, and the military health system, acting as “first movers,” can generate sufficient momentum to achieve consensus on value‐based payment and overcome inertia favoring volume‐based incentives.

Finally, targeted public and private investment in technologies and systems required to integrate information and achieve interoperability among disparate health information systems is an absolute requisite for enabling value‐based payment incentives to be translated into action along the continuum of patient care. Current stages of meaningful use in electronic health record incentives and certification provide a useful policy implementation framework for information support needed to drive value‐based payment: data capture and sharing, advancing clinical processes, and improving outcomes. Real‐time, clinically and managerially actionable data at these three levels of utility would create a technical platform for value‐based payment and care. Without fundamental change in health information technology that unifies clinical and economic data for use at the point of care, value‐based payment will be limited to marginal adjustments to a FFS, encounter‐driven health care system.
